# Propofol and α2-Agonists Attenuate Microglia Activation and Restore Mitochondrial Function in an In Vitro Model of Microglia Hypoxia/Reoxygenation

**DOI:** 10.3390/antiox11091682

**Published:** 2022-08-28

**Authors:** Lucia Longhitano, Alfio Distefano, Paolo Murabito, Marinella Astuto, Anna Nicolosi, Giovanni Buscema, Filippo Sanfilippo, Giuseppe Lazzarino, Angela Maria Amorini, Andrea Bruni, Eugenio Garofalo, Daniele Tibullo, Giovanni Li Volti

**Affiliations:** 1 Department of Biomedical and Biotechnological Sciences, University of Catania, Via S. Sofia 97, 95125 Catania, Italy; 2 Unità Operativa Complessa Anestesia e Rianimazione 2, Azienda Universitaria “Policlinico G. Rodolico” Via S. Sofia 97, 95125 Catania, Italy; 3 Azienda Ospedaliera “Cannizzaro”, Via Messina 628, 95126 Catania, Italy; 4 Anesthesia and Intesive Care Unit, Department of Medical and Surgical Sciences, University Hospital Mater Domini, Magna Grecia University, 88100 Catanzaro, Italy

**Keywords:** microglia, inflammation: mitochondria, hypoxia, propofol and α2-agonists

## Abstract

Cerebrovascular ischemia is a common clinical disease encompassing a series of complex pathophysiological processes in which oxidative stress plays a major role. The present study aimed to evaluate the effects of Dexmedetomidine, Clonidine, and Propofol in a model of hypoxia/reoxygenation injury. Microglial cells were exposed to 1%hypoxia for 3 h and reoxygenated for 3 h, and oxidative stress was measured by ROS formation and the expression of inflammatory process genes. Mitochondrial dysfunction was assessed by membrane potential maintenance and the levels of various metabolites involved in energetic metabolism. The results showed that Propofol and α2-agonists attenuate the formation of ROS during hypoxia and after reoxygenation. Furthermore, the α2-agonists treatment restored membrane potential to values comparable to the normoxic control and were both more effective than Propofol. At the same time, Propofol, but not α2-agonists, reduces proliferation (Untreated Hypoxia = 1.16 ± 0.2, Untreated 3 h Reoxygenation = 1.28 ± 0.01 vs. Propofol hypoxia = 1.01 ± 0.01 vs. Propofol 3 h Reoxygenation = 1.12 ± 0.03) and microglial migration. Interestingly, all of the treatments reduced inflammatory gene and protein expressions and restored energy metabolism following hypoxia/reoxygenation (ATP content in hypoxia/reoxygenation 3 h: Untreated = 3.11 ± 0.8 vs. Propofol = 7.03 ± 0.4 vs. Dexmedetomidine = 5.44 ± 0.8 vs. Clonidine = 7.70 ± 0.1), showing that the drugs resulted in a different neuroprotective profile. In conclusion, our results may provide clinically relevant insights for neuroprotective strategies in intensive care units.

## 1. Introduction

Cerebrovascular ischemia is a notably common clinical cerebrovascular disease encompassing a series of complex pathophysiological processes. Oxidative/nitrosative stress and inflammation are considered the key players of such injury, activating the crosstalk among different cell populations of the brain, such as microglia and neurons [1]. In recent decades, various pharmacological agents targeting neuroinflammation, apoptosis, and oxidative/nitrosative stress have been proposed as neuroprotectants in various clinical protocols [2]. The inhibition of the aforementioned molecular processes is essential to decrease neuronal injury and to obtain a concomitant improvement in the outcome and recovery of the patients admitted to the intensive care units (ICU). Clinically, an inadequate sedative technique may adversely affect morbidity and even mortality in the ICU since the sedative protocol modulates the neuroendocrine stress and the inflammatory response to surgery, thus significantly affecting the recovery period. In this regard, Dexmedetomidine is largely used in neuroanaesthesiology for the maintenance of haemodynamic stability, preserving intracranial homeostasis, reducing cerebral blood flow, and inducting neuroprotective effects [3,4,5]. Such a pharmacological profile allows a better evaluation of the neurological status of the patients in mechanical ventilation, especially when compared with other sedatives used in ICUs. Interestingly, Dexmedetomidine’s neuroprotective effects seem related both to its α2 adrenergic receptor agonism and to its binding at imidazoline 1 and 2 receptors [6]. In particular, Dexmedetomidine decreased lactate dehydrogenase release from mouse cortical neuronal cultures exposed to oxygen–glucose deprivation and reduced the infarct area and neurologic deficits in rats subjected to focal ischemia [6]. Previous studies focused on the biochemical mechanisms underlying Dexmedetomidine’s neuroprotection showed that Dexmedetomidine decreased the pro-apoptotic Bcl2-associated protein expression and increased the anti-apoptotic Bcl2 protein expression in the hippocampus of rats undergoing incomplete cerebral ischemia [7,8]. On the other hand, the use of other α2 adrenergic receptor agonists (i.e., Clonidine) failed to demonstrate neuroprotection in focal cerebral ischemia in WT, (2A)(-/-), α(2B)(-/-), and α(2C)(-/-) mice. By contrast, in a severe hypoxic preterm fetal sheep model, Clonidine administration shortly after perinatal hypoxia-ischemia promoted neural recovery but highlighted the complex dose–response of exogenous therapy.

Sedative agents other than α(2)-adrenoceptors agonists have widely been used and exploited for their neuroprotective profile. In this regard, Propofol has been administered in pediatric anesthesia for many years due to its attractive pharmacokinetic profile, rapid onset of pharmacological effects, and the short recovery period in post-anesthetized patients [9,10,11]. As for many anesthetics, the exact mechanism of action of Propofol has never been fully elucidated. Several reports indicate that the anesthetic effects of Propofol are mediated by potentiation of the GABAA receptor [12]. Contrary to Dexmedetomidine, data relevant to the neuroprotective effects of Propofol are contradictory. Similarly to Dexmedetomidine, Propofol increased Bcl2 and decreased Bcl2-associated protein three days after cerebral ischemia and reperfusion in the hippocampus [13].

Based on the aforementioned findings, the present study aimed to evaluate and compare the effects of Dexmedetomidine, Clonidine (two α(2)-adrenoceptor agonists), and Propofol on oxidative/nitrosative stress, inflammation, and energy metabolism in hypoxia-activated microglia.

## 2. Materials and Methods

### 2.1. Cell Culture, Induction of Hypoxia/Reoxygenation and Pharmacological Treatments

The BV2 cells (mouse microglial cells) were purchased from ATCC Company (Milan, Italy). The cells were suspended in DMEM (Dulbecco’s Modified Eagle Medium containing 10% fetal bovine serum (FBS), 100 U/mL of penicillin, and 100 U/mL of streptomycin) culture medium. At 80% confluency, the cells were treated with trypsin-EDTA solution (0.05% trypsin and 0.02% EDTA), and the resulting cell suspension was adjusted to a cell density of 5 × 104 cells/mL. The cell suspensions were washed with PBS (Phosphate-Buffered Saline), resuspended in serum-free DMEM medium, and placed in an incubator at 37 °C, without and with the addition of Propofol, Dexmedetomidine, or Clonidine (Sigma–Aldrich, Milan, Italy). The drugs were tested at final concentrations of 25 and 50 μM. Hypoxia was then induced by challenging the cell cultures with a gas mixture containing 1.0% O2, 5% CO2, and 37 °C for 3 h to initiate hypoxia, followed by 3 h of reoxygenation at 37 °C using a gas mixture containing 5% CO2 and 18.0% O2. We used gas-controlled incubators to control the O2 levels of the cell cultures. In hypoxia, the O2 levels were 1%, and the CO2 levels were 5%. Under the reoxygenation conditions, the O2 levels were 18%, and the CO2 levels were 5%. Nitrogen was added to the incubator in order until the set O2% was achieved.

### 2.2. Confocal Microscopy for the Evaluation of Mitochondrial Membrane Potential (Δψ)

Mitochondrial Δψ was assessed in living cells using the fluorescent intensity of the JC-1 probe (10 μg/mL) [14] at emission wavelengths of 585 nm (red fluorescence = high value of Δψ) and 527 nm (green fluorescence = low value of Δψ). JC-1 permeates the mitochondria as a function of Δψ, giving a red fluorescence when the mitochondrial membrane potential is high and shifting into green fluorescence when the mitochondrial membrane potential is decreased.

Briefly, the cells were seeded in a 96-well multiplate (Cell carrier ultra) at a density of 5 × 10^3^ cells. After 24 h, the cells were exposed to hypoxia (3 h) and reoxygenation and incubated with media containing dye for 1 h. Then, the cells were washed and read in confocal conditions using the 20× long WD objective by the High Content Screening (HCS) analysis system (PerkinElmer Operetta High-Content Imaging System) for 24 h.

### 2.3. RNA Extraction and RT-qPCR

cDNA was synthesized with a High-Capacity cDNA Reverse Transcription kit (category no. 4368814, Applied Biosystems, Foster City, CA, USA) and the quality was checked, taking into consideration the housekeeping gene Ct values. Quantitative real-time PCR was performed in a QuantStudio™ 3 Real-Time PCR system, Applied Biosystems, using the SYBR Green PCR MasterMix (category no. 4309155, Life Technologies, Monza, Italy). The primers were designed using BLAST^®^ (Basic Local Alignment Search Tool, NBCI, NIH). The primers’ sequences are shown in Table 1, and β-actin was used as the housekeeping gene. The relative mRNA expression level was calculated by the threshold cycle (Ct) using a comparative 2^−ΔΔCt^ method.

### 2.4. Real-Time Monitoring of Cell Proliferation

xCELLigence experiments were performed using the RTCA (Real-Time Cell Analyzer) DP (Dual Plate) instrument according to the manufacturers’ instructions (Roche Applied Science, Mannheim, Germany, and ACEA Biosciences, San Diego, CA). E-plate16 is a single-use 16-well cell culture plate with bottom surfaces covered with microelectrode sensors (0.2 cm2 well surface area; 243 ± 5 μL maximum volume). Real-time changes were expressed as a *cell index* defined as (Rn-Rb)/15, where Rb is the background impedance, and Rn is the impedance of the well with cells. The negative control groups were tested in every experiment. Before seeding the cells, the background impedance was measured after the 30 min incubation period at room temperature. After seeding 5000 cells into each well, the plate was incubated at room temperature for 30 min, and cell proliferation was monitored every 20 min for 24 h.

### 2.5. Intracellular ROS Measurement

To determine intracellular ROS generation, we stained the cells with 5 mM dihydroethidium (DHE, Sigma-Aldrich, Milan, Italy) in PBS for 30 min at 37 °C. Fluorescence intensity (excitation at 488 nm, emission at 620 nm) was measured by fluorescence-activated cell sorting (FACS, FC500, Beckman Coulter, Milan, Italy) [15].

### 2.6. Effects of Pharmacological Treatments on Cell Migration

Cell migration was performed using a wound healing assay. Briefly, the cells were seeded in 24-well dishes, and the cells were then scraped with a 200 μL micropipette tip. After the different drug treatments, the cells were monitored at 0 h, 4 h, 8 h, 12, 18 h, and 24 h and placed in Operetta (Perkin Elmer) at 37 °C and 5% CO2. The wound area was measured and quantified at different intervals using Harmony high-content imaging and analysis software (PerkinElmer, MA, USA) following cell segmentation.

### 2.7. Cytokine Antibody Arrays

To determine the cytokine concentrations of a total of 40 inflammatory factors, we used a Mouse Inflammation Q1 kit (Raybiotech Inc., Georgia, USA, www.raybiotech.com, 1 April 2021) according to the manufacturer’s instructions. Multiple cytokine-specific capture antibodies were first bound to glass surfaces, and following incubation with the samples, the target cytokines concentration was by InnoScan 700/710 Microarray Scanner (Innopsys) for quantitative analysis.

### 2.8. HPLC Analysis of Metabolites

The packed cells were deproteinized to measure acid labile and easily oxidizable compounds [16]. The simultaneous separation of high-energy phosphates (ATP, ADP, AMP, GTP, GDP, GMP, IMP, UTP, UDP, UMP, CTP, CDP, CMP), Coenzyme A and its derivatives (Acetyl-CoA, Malonyl-CoA), nicotinic coenzymes (NAD+, NADH, NADP+, NADPH), reduced glutathione (GSH), malondialdehyde (MDA), nitrite, and nitrate in the protein-free cell extracts, was carried out using the established HPLC methods [16,17,18].

### 2.9. Statistical Analysis

The results are expressed as the means ± standard deviation (SD) of at least three independent experiments. Statistical analysis was carried out by one-way analysis of variance using the GraphPad Prism 8.01 software (GraphPad Software, San Diego, CA, USA). Differences were considered significant at *p* < 0.05.

## 3. Results

### 3.1. Propofol and α_2_-Agonists Attenuate Reactive Oxygen Species Formation during Hypoxia and Following Reoxygenation

**Figure 1:**
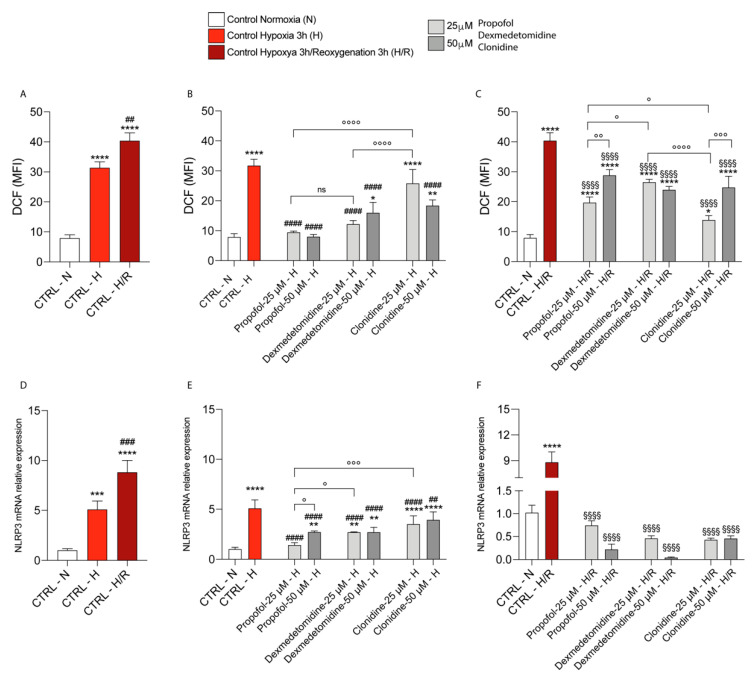
Effect of hypoxia and reoxygenation (**A**) on ROS production in microglia cells. Effect of Propofol, Dexmedetomidine, and Clonidine on ROS production after 3 h of Hypoxia (**B**) and 3 h of Reoxygenation (**C**), analyzed by flow cytometric assay. Data are expressed as mean ± SD of at least four independent experiments. * vs. Untreated Normoxia; (* *p* < 0.05; ** *p* < 0.005; *** *p* < 0.001; **** *p* < 0.0001); # vs. Untreated Hypoxia; (^##^ *p* < 0.005; ^###^ *p* < 0.001; ^####^ *p* < 0.0001) § vs. Untreated Reoxygenation (^§§§§^ *p* < 0.0001) ° vs. the different drugs (propofol vs. dexmedetomidine; propofol vs. clonidine; dexmedetomidine vs. clonidine) (° *p* < 0.05; °° *p* < 0.005; °°° *p* < 0.001; °°°° *p* < 0.0001). Effect of Hypoxia and Reoxygenation on NLRP3 mRNA expression in (**D**) Untreated cells, and Propofol, Dexmedetomidine, and Clonidine treated cells (**E**,**F**), analyzed by real-time PCR. The calculated value of 2^−ΔΔCt^ in untreated controls is 1. Data are expressed as mean ± SD of at least four independent experiments. * vs. Untreated Normoxia ( ** *p* < 0.005; *** *p* < 0.001; **** *p* < 0.0001); # vs. Untreated Hypoxia (^##^ *p* < 0.005; ^###^ *p* < 0.001; ^####^ *p* < 0.0001); § vs. Untreated Reoxygenation (^§§§§^ *p* < 0.0001); ° vs. the different drugs (propofol vs. dexmedetomidine; propofol vs. clonidine; dexmedetomidine vs. clonidine) (° *p* < 0.05; °°° *p* < 0.001).

We firstly aimed to study the effect of hypoxia and reoxygenation on ROS formation. Our results showed that the hypoxia (3 h)-treated cells resulted in a significant increase in ROS formation when compared to the normoxic cells (Figure 1A). Furthermore, the hypoxia/reoxygenation (3 h)-treated cells resulted in a further significant increase in ROS formation (Figure 1A) when compared to normoxia and hypoxia. Under hypoxic conditions, the cells exposed to Propofol and α2-agonists (Dexmedetomidine and Clonidine) showed a significant decrease in ROS formation when compared to the untreated hypoxic cell cultures (Figure 1B). In this condition, there are no significant differences between the cells treated with Propofol and Dexmedetomidine (both concentrations of 25 and 50 µM), while their effect on ROS production is more significantly reduced compared to the treatment with Clonidine at the final concentration of 25 µM (Figure 1B). Likewise, all of the drug treatments produced a significant decrease in ROS formation during reoxygenation compared to the hypoxia/reoxygenation-treated cells (Figure 1C). Interestingly, the decrease in ROS production determined by Propofol 25 µM was significantly higher compared to both Propofol 50 µM and Dexmedetomidine 25 µM, although Clonidine 25 µM had the most relevant effects on ROS production compared to both Propofol and Dexmedetomidine (Figure 1C).

Consistently, our results showed that, in untreated cells, hypoxia and consequent reoxygenation induced a significant increase in NLRP3 mRNA expression levels (Figure 1D), and that, under hypoxia, all three treatments induced a significant decrease in NLRP3 mRNA expression levels, compared to untreated cells (Figure 1E). In this case, the highest effect on NLRP3 mRNA expression was produced by Propofol 25 µM compared to Dexmedetomidine and Clonidine 25 µM (it is worthy of note that none of the drugs had a significant effect on NLRP3 expression under normoxia, Figure S1). The effect of the three drugs in reducing NLRP3 mRNA expression levels was even more evident in cells exposed to hypoxia/reoxygenation (compared to both the control cells and the untreated hypoxia/reoxygenation cells), even though no significant differences between the three pharmacological treatments were observed (Figure 1F).

### 3.2. Propofol and α2-Agonists Restore Mitochondrial Membrane Potential (Δψ)

**Figure 2:**
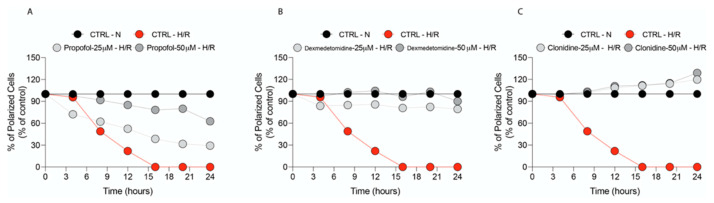
Real-time analysis of ΔΨm modification. JC-1 accumulates in mitochondria as a function of Δψ is excited at 490 nm, and emits at 527 nm when in monomeric form. At high Δψ, JC-1 is concentrated within mitochondria and forms J aggregates, resulting in a shift in emission to 585 nm. Real-time analysis of ΔΨm modification monitoring by Operetta Hugh Content Screening following treatment with (**A**) Propofol, (**B**) Dexmedetomidine, and (**C**) Clonidine. Results are presented as the mean ± SD of four independent experiments.

Figure 2A–C shows that the untreated normoxic cells had a large number of green, fluorescent mitochondria, representing J aggregates that accumulate at normally hyperpolarized membrane potential. Hypoxic cells showed fewer red J aggregates, indicating a gradual dissipation of Δψm (for a better understanding of the Δψm trend, we show our data in % control, assigning 100% polarized cells to normoxic cells at each time point analyzed). Following hypoxia/reoxygenation, a rapid and significant reduction of % polarized cells compared to normoxic control cells took place (Figure 2A–C).

Significant recovery of Δψm were obtained by the treatment with any of the selected drugs at both doses tested (interestingly, none of the drugs had a significant reduction effect on the % of polarized cell in normoxia, Figure S2). However, the treatments with Dexmedetomidine and Clonidine restored Δψm to values comparable to those of the normoxic control and were both more effective than Propofol.

### 3.3. Propofol but Not α2-Agonists Reduces Microglia Cell Proliferation and Migration Following Hypoxia and Reoxygenation

**Figure 3:**
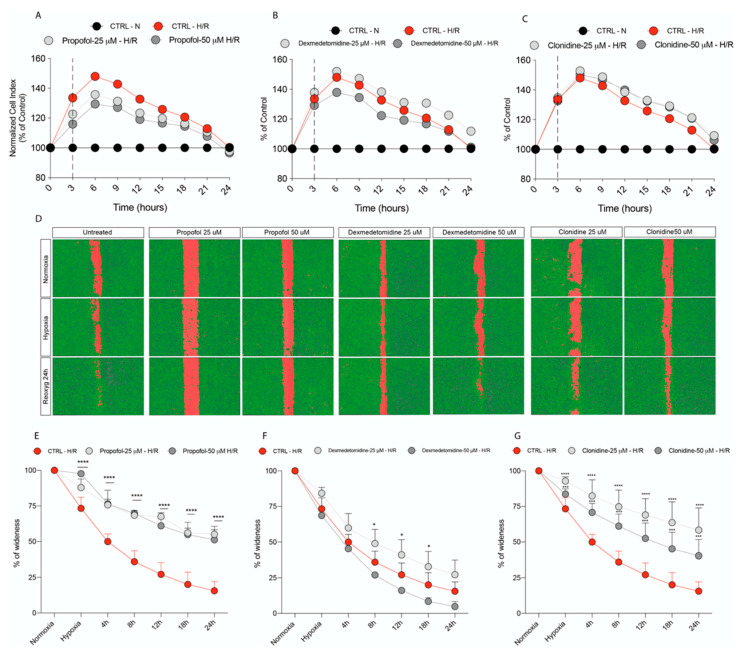
Real-time cell proliferation monitoring by xCELLigence system following treatments with (**A**) Propofol, (**B**) Demedetomidine, and (**C**) Clonidine, after Hypoxia (3 h) and Reoxygenation. Cell index values were normalized at the time of onset of hypoxia in order to obtain a normalized cell index. The value of normalized cell index of each treatment was normalized in % of normoxic control. Each line is expressing the average of four different experiments. (**D**) Cell migration analysis following treatments with (**E**) Propofol, (**F**) Dexmedetomidinel and (**G**) Clonidine, analyzed by Operetta High Content Screening. Values are presented as percentage of the open wound following Hypoxia (3 h) and 4, 8, 12, 18, and 24 h of Reoxygenation (wound at time 0 was assumed as 100% and used as control). Values are expressed as the mean ± SD of four different experiments. * vs. Untreated. (* *p* < 0.05; *** *p* < 0.001; **** *p* < 0.0001).

Our results showed a significant increase in cell proliferation during hypoxia and reoxygenation as a result of microglia activation, as measured by an increased cell index normalized to normoxic control (Figure 3A–C and Table 2). Interestingly, Propofol (50 μM) resulted in a significant decrease in cell proliferation during hypoxia and 3 h of reoxygenation (Figure 3A and Table 2) when compared to the untreated hypoxic cell cultures, whilst the two other treatments were ineffective at both doses tested (Figure 3B,C and Table 2). Our results also showed that Propofol and Clonidine 25 µM and 50 µM significantly inhibited cell migration compared to the control cells (Figure 3D,F). In comparison, Dexmedetomidine was only effective when the 25 µM concentration was used (Figure 3E).

### 3.4. Propofol and α2-Agonists Reduce Inflammatory Gene and Protein Expressions during Hypoxia and Following Reoxygenation

**Figure 4:**
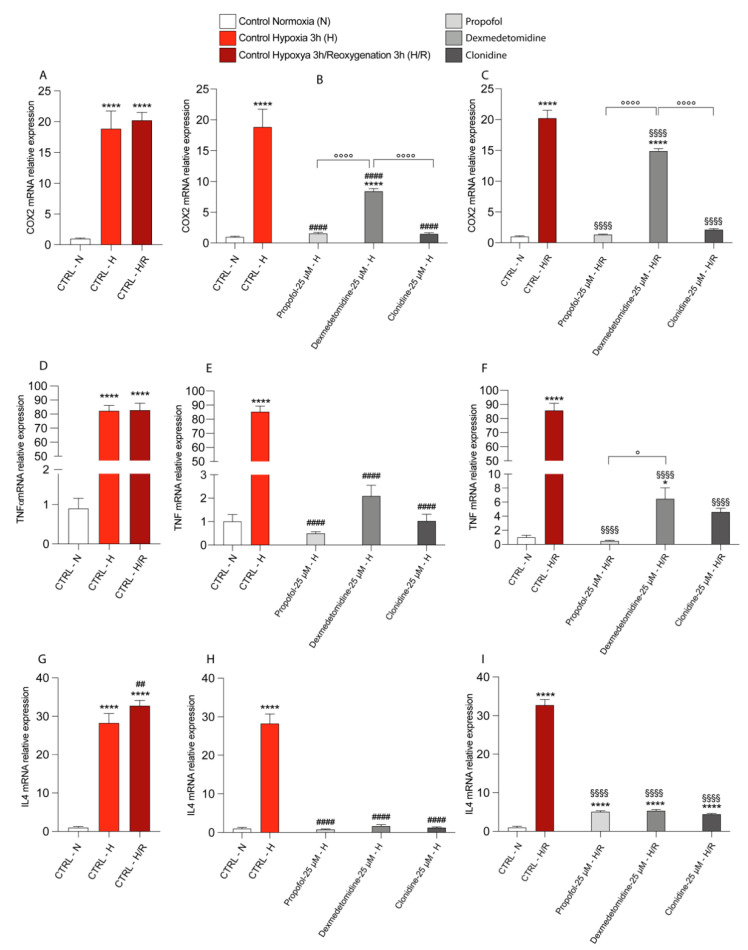
Effect of Hypoxia and Reoxygenation and pharmacological treatments on mRNA expression of COX2 (**A**–**C**), TNF (**D**–**F**), IL4 (**G**–**I**), performed by Real-time PCR. The calculated value of 2^−^^ΔΔCt^ in untreated controls is 1. Data are expressed as mean ± SD of at least four independent experiments. * vs. CTRL Normoxia (* *p* < 0.05; **** *p* < 0.0001); # vs. CTRL Hypoxia (^##^ *p* < 0.005; ^####^ *p* < 0.0001); § vs. CTRL Hypoxya/reoxygenation (^§§§§^ *p* < 0.0001); ° vs. the different drugs (propofol vs. dexmedetomidine; propofol vs. clonidine; dexmedetomidine vs. clonidine) (° *p* < 0.05; °°°° *p* < 0.0001).

Since in the majority of the experiments described up to now, the lower dose of each drug tested was that producing the most significant beneficial effects, we chose the use of the 25 μM concentration only in the experiments described hereinafter. As shown in Figure 4A,D,G, hypoxia and the consequent reoxygenation in untreated cells caused a significant increase in the mRNA levels of COX2 (Figure 4A), TNF (Figure 4D), and IL4 (Figure 4G) compared to the values measured in the normoxic untreated cells. The treatment with either Propofol, Dexmedetomidine, or Clonidine induced a significant decrease in the mRNA expressions of COX2 (Figure 4B), TNF (Figure 4E), and IL4 (Figure 4H) under hypoxic conditions compared to the hypoxic untreated cells. Similar effects were obtained under hypoxia/reoxygenation conditions (Figure 4C,F,I). In both experimental conditions (hypoxia and hypoxia/reoxygenation), Propofol and Clonidine had the most relevant effects in decreasing the mRNA expression levels of COX2 (Figure 4B,C), and Propofol also significantly decreased TNF expression (Figure 4F). No differences among the three pharmacological treatments were observed when considering the gene expression of IL4 (Figure 4H,I).

**Figure 5:**
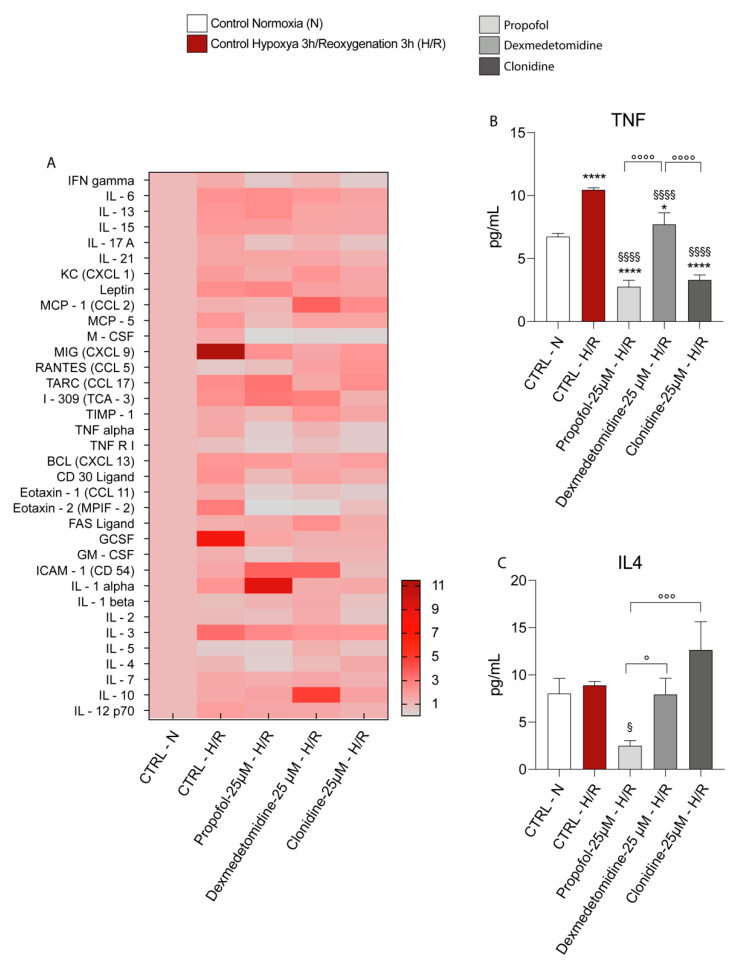
(**A**) Heatmap representing the levels of major cytokines and chemokines detected Cytokine antibody arrays. (**B**) TNF, (**C**) IL4 levels. Results are presented as the mean ± SD of four independent experiments. * vs. CTRL Normoxia (* *p* < 0.05; **** *p* < 0.0001); § vs. CTRL Hypoxia/Reoxygenation. (^§^ *p* < 0.05; ^§§§§^ *p* < 0.0001); ° vs. the different drugs (propofol vs. dexmedetomidine; propofol vs. clonidine; dexmedetomidine vs. clonidine) (° *p* < 0.05; °°° *p* < 0.001; °°°° *p* < 0.0001).

In order to confirm the RT-PCR data, we performed a protein array for cytokine and chemokine detection and quantification. In Figure 5A, we reported a heatmap representing all the changes in the levels of cytokines and chemokines, showing that 3 h of 1% of hypoxia followed by 3 h of reoxygenation significantly increased a plethora of cytokines and chemokines compared to the normoxic control; in particular, Figure 5B,C, respectively, confirmed the RT-PCR data only in the case of TNF protein expression (increase in the expression level), since the protein expression of IL4 did not undergo significant changes compared to the normoxic control. The three drugs under evaluation significantly decreased TNF production (Figure 5B) under both normoxia and hypoxia/reoxygenation conditions, with Propofol and Clonidine provoking a significant decrease in TNF production compared to Dexmedetomidine (Figure 5A,B). Interestingly, IL4 protein levels were found significantly lower than those measured in normoxic controls and untreated hypoxia/reoxygenation cells, as well as in hypoxia/reoxygenation cells challenged with Dexmedetomidine and Clonidine, only by Propofol treatment (Figure 5C).

**Figure 6:**
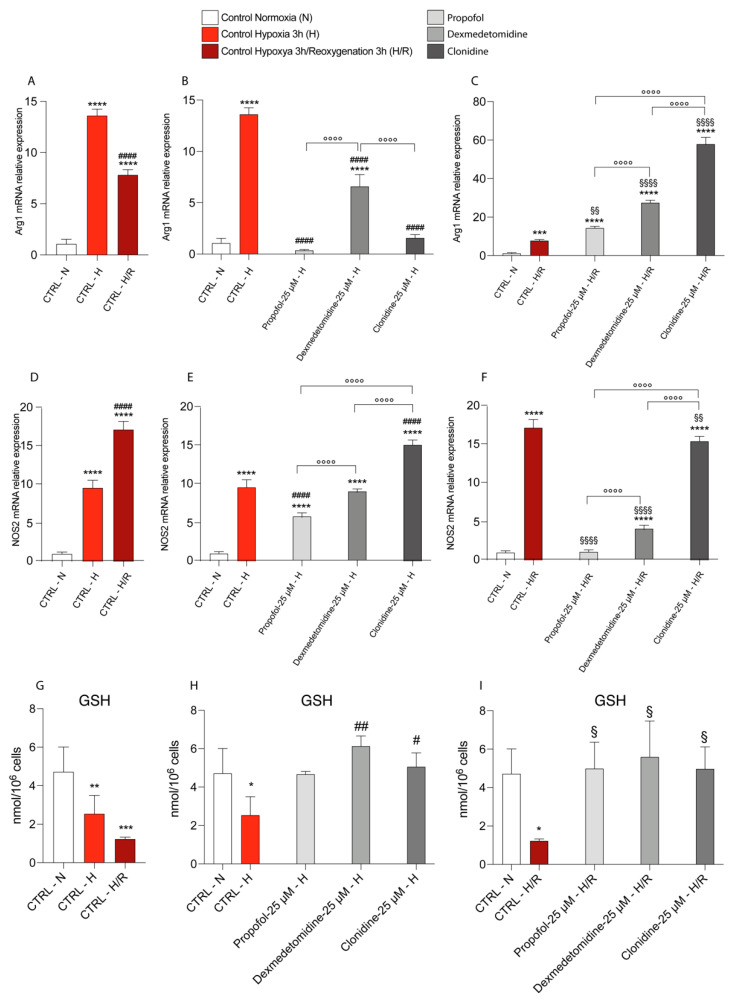
Effect of Hypoxia and Reoxygenation and pharmacological treatments on mRNA expression of Arg1 (**A**–**C**) and NOS2 (**D**–**F**), performed by Real-time PCR. The calculated value of 2^−ΔΔCt^ in untreated controls is 1. GSH levels detected by HPLC (**G**–**I**). Data are expressed as mean ± SD of at least four independent experiments. * vs. CTRL Normoxia; (* *p* < 0.05; ** *p* < 0.005; *** *p* < 0.001; **** *p* < 0.0001); # vs. CTRL Hypoxia; (^#^ *p* < 0.05; ^##^ *p* < 0.005; ^####^ *p* < 0.0001); § vs. CTRL Hypoxya/reoxygenation (^§^ *p* < 0.05; ^§§^ *p* < 0.005; ^§§§§^ *p* < 0.0001); ° vs. the different drugs (propofol vs. dexmedetomidine; propofol vs. clonidine; dexmedetomidine vs. clonidine) (°°°° *p* < 0.0001).

We also assessed NOS2 and Arg1 gene expressions to evaluate the effect of hypoxia/reoxygenation and treatments on microglial activation. Significant increases in NOS2 and Arg1 expressions in hypoxia untreated cells were determined compared to the levels measured in the normoxic untreated cells (Figure 6A,B). Reoxygenation (3 h) resulted in a slight decrease (compared to hypoxic control cells) in Arg1 gene expressions (Figure 6A) and a further dramatic increase in NOS2 gene expressions (Figure 6B).

Among the three drugs under study, only Propofol effectively counteracted the increase in NOS2 expressions both after hypoxia (Figure 6E) and hypoxia/reoxygenation (Figure 6F), whilst Dexmedetomidine was modestly effective only in hypoxic cells and Clonidine modestly effective only in hypoxic hypoxia/reoxygenation cells (Figure 6E,F). By contrast, when measuring Arg1 mRNA levels under hypoxic conditions, each drug variably decreased this gene expression compared to untreated hypoxic control cells and variably increased mRNA levels compared to the hypoxia/reoxygenation control cells (Figure 6C). These data suggest that both hypoxia and hypoxia/reoxygenation promote the inflammatory phenotype of microglia, and that was variously influenced by pharmacological treatments.

These results were confirmed by measuring GSH levels by HPLC. In particular, the data show that under hypoxia and hypoxia/reoxygenation conditions, there is a significant reduction in GSH levels in the untreated cells (Figure 6G) and that these levels are restored by pharmacological treatments both in conditions of hypoxia (Figure 6H) and in conditions of hypoxia/reoxygenation (Figure 6I); however, there are no significant differences between the three treatments.

### 3.5. Propofol and α2-Agonists Restore Energy Metabolism of Microglia Cells

**Figure 7:**
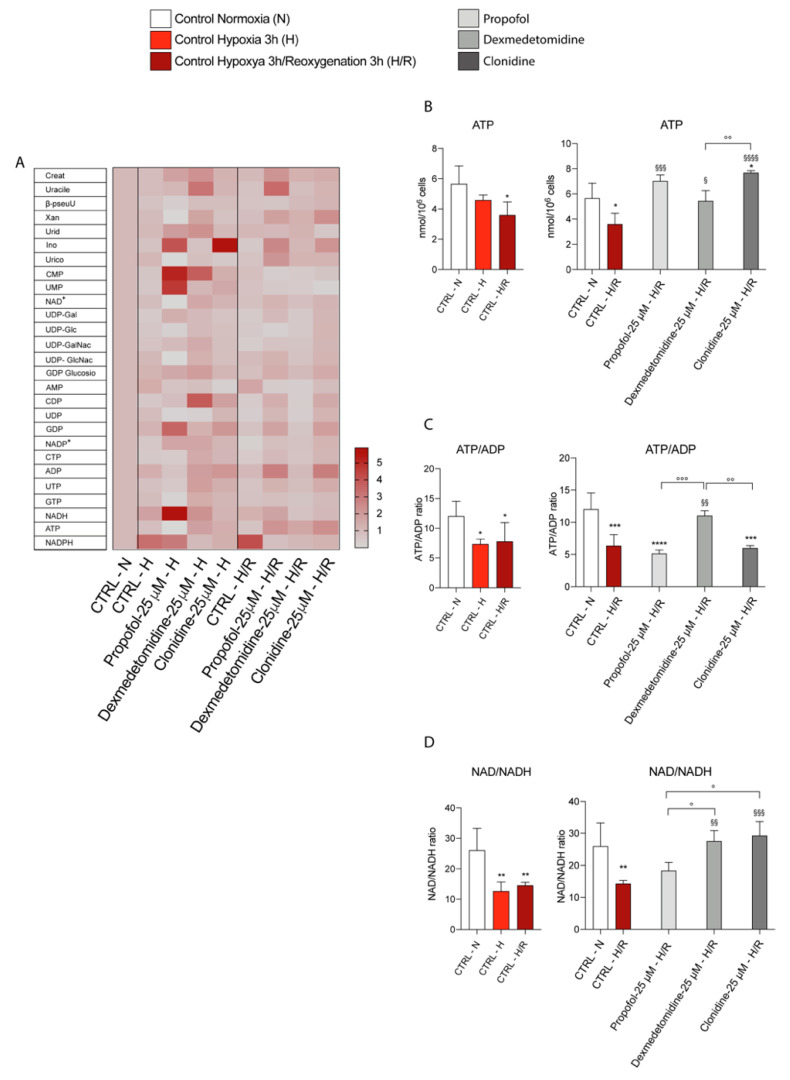
(**A**) Heatmap representing the levels of major classes of metabolites detected by HPLC. (**B**) ATP levels; (**C**) ATP/ADP levels; (**D**) NAD/NADH levels. Results are presented as the mean ± SD of four independent experiments. * vs. CTRL Normoxia (* *p* < 0.01; ** *p* < 0.005; *** *p* < 0.001); § vs. CTRL Hypoxya/reoxygenation (^§^ *p* < 0.05; ^§§^ *p* < 0.005; ^§§§^ *p* < 0.001; ^§§§§^ *p* < 0.0001); ° vs. the different drugs (propofol vs. dexmedetomidine; propofol vs. clonidine; dexmedetomidine vs. clonidine) (° *p* < 0.05; °° *p* < 0.005; °°° *p* < 0.001).

To assess the impact of hypoxia and reoxygenation on cellular energy metabolism, we further analyzed the endogenous metabolic profiles of the control, hypoxia/reoxygenation, and treated cells (under both normoxia and hypoxia/reoxygenation). In Figure 7A, we reported a heatmap representing changes in the metabolites representative of energy metabolism and mitochondrial function. The results showed that hypoxia/reoxygenation (3 h) significantly decreased ATP content (Figure 7B) compared to the normoxic cells; each of the three treatments induced a significant increase in ATP content (Figure 7A,B) compared to both normoxia and hypoxia untreated cells, suggesting that Propofol and α2–agonist are able to restore ATP levels during hypoxia/reoxygenation. At the same time, the ATP/ADP ratio showed a tendency to be lower in untreated cells (Hypoxia/reoxygenation), and this effect was counteracted only by Dexmedetomidine treatment (Figure 7C). Consistent with this, the NAD+/NADH ratio (Figure 7D) decreased in hypoxia/reoxygenation cells compared to the normoxic cells and differentially increased when Propofol, Dexmedetomidine, or Clonidine were added to the cell medium (Figure 7D).

## 4. Discussion

The α2-Adrenoceptor agonists Dexmedetomidine and Clonidine and/or Propofol are routinely used in ICUs as sedative agents exerting significant clinical neuroprotective effects [12,19,20]. Cerebral I/R leads to increased oxidative stress, triggering a series of molecular events (i.e., inflammation and mitochondrial dysfunction) and leading to brain injury. In particular, microglia respond to damage-associated molecular patterns (DAMPs) by increasing the production of ROS responsible for oxidative injury [21]. Consistently, our results showed that hypoxia/reoxygenation resulted in a significant increase in ROS generation compared to normoxic cells. Such evidence is of great clinical relevance since ROS production, oxidative stress, and subsequent mitochondrial dysfunction are determinants during reperfusion, in causing I/R injury but may be impacted by different anesthetic agents [22]. Notably, Propofol, an intravenous anesthetic agent, chemically similar to free radical scavengers such as alpha tocopherol, has been associated with reduced damage and apoptosis in I/R injury [23,24]. Similarly, Holownia et al. [25] showed that in vitro, Propofol restored glutamine synthase activity, decreased intracellular ROS, and protected rat astrocytes from t-BOOH-induced apoptosis. In an in vitro model of oxidative stress [26], 6 h of oxygen and glucose deprivation (OGD) followed by 24 h of reoxygenation caused the widespread cell death of rat astrocytes which was prevented by treatment with Propofol before OGD/reoxygenation [26,27]. Consistent with these observations, our data, in an in vitro model of microglial cells, showed that Propofol was able to reverse ROS production both under hypoxic conditions and the following reoxygenation. The same results were obtained by treatment with α2-agonists Dexmedetomidine and Clonidine. Consistently, Syamimi et. Al showed that Clonidine treatment elevated total antioxidant status and decreased the level of lipid peroxidation and protein oxidation products in heart [28].

Several studies reported that the ROS generated during mitochondrial dysfunction, as measured by a decrease in mitochondrial membrane potential (∆ψm), promote NLRP3 inflammasome activation [29]. Interestingly, the Propofol and Dexmedetomidine treatment showed significant restoration of Δψm following 3 and 24h of reoxygenation compared to untreated cells; whilst Clonidine was ineffective. In addition, ROS generation and subsequent mitochondrial damage trigger the formation and activation of microglial NLRP3 inflammasome [29,30,31,32], leading to the abundant secretion of IL-1β [33]. In prior studies, Dexmedetomidine treatments have been shown to alleviate hyperoxia-induced acute lung injury and liver injury through the inhibition of the activation of NLRP3 inflammasomes [34,35]. Similarly, Propofol has been shown to provide liver protection against D-GalN/LPS-induced liver damage in mice by inhibiting oxidative stress, inflammation, and hepatocyte apoptosis by regulating the TLR4/NF-κB/NLRP3 pathway [36]. NLRP3 inflammasome activation is known to be an indicator of the development of neurodegenerative diseases [37,38,39,40,41] and is also a pre-requisite for neuroinflammation initiation [42,43]. Thus, we hypothesized that the protective effects of the tested compounds might be related to the inhibition of NLRP3 inflammasome activation. Interestingly, during the hypoxic phase, we observed a significant increase in NLRP3 mRNA expression levels and that both Propofol and both alpha 2 agonists significantly prevented NLRP3 expression during the reoxygenation phases. In particular, both concentrations of Propofol, Dexmedetomidine, and Clonidine tested were able to restore NLRP3 expression to control levels during the reoxygenation phase at 3 and 24 h.

Our results showed that hypoxia induced a significant increase in microglia proliferation peaking at 3 h of oxygen decrease, which was resolved following 24 h of reoxygenation. Interestingly, α2-agonists did not modify the proliferative profile of microglia, whereas the highest dose of Propofol resulted in a significant reduction in the proliferative peak. Furthermore, microglia can modify their migratory capacity when stimulated by a hypoxic insult, moving towards the site of the lesion [44], releasing not only inflammatory cytokines but also chemokines.

In response to various brain injuries, microglia are activated and polarized into the proinflammatory (M1-like phenotype) or the anti-inflammatory (M2-like phenotype) [45]. Thus, we hypothesized that the tested drugs were able to promote M2 activation of microglia. Our results demonstrated that hypoxia and reoxygenation, in the absence of drug treatment, induced a significant increase in mRNA levels of M1 and M2 markers. On the contrary, both Propofol and α2-agonists induce the switch towards the anti-inflammatory M2 phenotype, thus facilitating recovery following the hypoxic insult.

Different microglia phenotypes are also related to differences in metabolic processes suggesting a role of mitochondrial activity in phenotypic outcomes [46]. Therefore, we studied the metabolites representative of energy metabolism and mitochondrial functions of cells, such as the levels of ATP, ADP, NAD+, and NADH. Our results demonstrated a reduction in ATP levels in hypoxic cells in relation to normoxic controls and also a significant decrease in the NAD+/NADH ratio [47]. Since mitochondria in hypoxia are dysfunctional, the lower formation of NAD+ and consequent increase in NADH are indicative of increased glycolytic rates to compensate for the decreased oxidative phosphorylation-produced ATP [48,49]. Interestingly, during reoxygenation, pharmacological Propofol and α2-agonists treatments restored ATP levels and NAD+/NADH ratio, confirming their protective role against hypoxic damage by ameliorating mitochondrial functions.

## 5. Conclusions

In conclusion, our results suggested that treatment with Propofol, Dexmedetomidine, and Clonidine exhibits different neuroprotective profiles and possible therapeutic window and play an important role in neuroprotection following hypoxic injury, representing valid pharmacological strategies for neuroprotection in critically ill patients.

## Figures and Tables

**Table 1:** Primer sequences of gene of interest.

Gene	Forward Primer (5′ ⟶ 3′)	Reverse Primer (5′ ⟶ 3′)	Access Number
*nlrp3*	TGCTCTTCACTGCTATCAAGCCCT	ACAAGCCTTTGCTCCAGACCCTAT	NM_145827.4
*cox2*	GATGACTGCCCAACTCCC	AACCCAGGTCCTCGCTTA	NM_011198.4
*arg1*	GCATATCTGCCAAAGACATCG	CCATCACCTTGCCAATCCC	NM_007482.3
*tnf*	CCCTTCCTCCGATGGCTAC	CGCCTCCTTCTTGTTCTGG	NM_001177759.1
*nos2*	GAGCGAGTTGTGGATTGTC	GGCAGCCTCTTGTCTTTG	NM_001313922.1
*il-4*	CAACCCCCAGCTAGTTGTCA	TGTCGCATCCGTGGATATGG	NM_021283.2
*β-actin*	CCTTCTGACCCATTCCCACC	GCTTCTTTGCAGCTCCTTCG	NM_007393.5

**Table 2:** Normalized cell Index raw data values. Normalized Cell Index raw data values during Hypoxia and different time of reoxygenation following various pharmacological treatments. * *p* < 0.0001 vs. Untreated Normoxia, ** *p* < 0.005 vs. Untreated Hypoxia.

	HYPOXIA	REOXYGENATION	
	0 h	3 h	24 h
Untreated Normoxia	0.87	0.87	1.31
Untreated Hypoxia	1.16	1.28 *	1.31
Propofol 25 μM	1.07	1.18	1.28
Propofol 50 μM	1.01 **	1.12 **	1.27
Dexmedetomidine 25 μM	1.20	1.32	1.47
Dexmedetomidine 50 μM	1.12	1.20	1.32
Clonidine 25 μM	1.17	1.32	1.43
Clonidine 50 μM	1.15	1.30	1.39

## Data Availability

All of the data are contained within the article and Supplementary Material.

## Supplementary Materials

The following supporting information can be downloaded at: https://www.mdpi.com/article/10.3390/antiox11091682/s1, Supplementary Materials and methods: HPLC analysis of metabolites; Figure S1: Effect of Propofol, Dexmedetomidine, and Clonidine treatments in the expression of genes under normoxic conditions; Figure S2: Real-time analysis of ΔΨm modification following Propofol, Dexmedetomidine, and Clonidine treatments under normoxic conditions.
