# Supplementary material for: Propofol and α2-Agonists Attenuate Microglia Activation and Restore Mitochondrial Function in an In Vitro Model of Microglia Hypoxia/Reoxygenation
Source: Antioxidants (Basel). 2022 Aug 28;11(9):1682. doi: 10.3390/antiox11091682 (PMC9495359; doi:10.3390/antiox11091682)
Supplement: Supplementary file 1 [file antioxidants-11-01682-s001.zip › antioxidants-1879550-supplementary.pdf]

Supplementary Materials and Methods

HPLC analysis of metabolites

The metabolic analysis was performed after deproteinization of cell samples (3x106 cells) according to a protocol suitable for obtaining protein-free extracts for further HPLC analysis of labile and easily oxidizable acid compounds. The cells were washed twice with PBS at pH 7.4 and collected by centrifugation at 1860 x g for 5 minutes at 4 ° C. Cell pellets were deproteinized by adding 1 ml of ice-cold, nitrogen-saturated KH2PO4, 10 mM in CH3CN, pH 7.4 (1: 3, v / v)[18]. After vigorous mixing for 60 seconds, the samples were centrifuged at 20 690 g for 10 minutes at 4 ° C. The organic solvent was removed from the deproteinized supernatants by two washes with 5 ml of chloroform. The upper aqueous phase obtained by centrifugation at the same conditions, was then used for the HPLC analysis of low molecular weight metabolites. Simultaneous separation of 50 low molecular weight metabolites related to energy metabolism, oxidative/nitrosative stress, antioxidants, and including high energy phosphates (ATP, ADP, AMP, GTP, GDP, GMP, UTP, UDP, UMP, CTP, CDP, CMP, IMP), oxidized and reduced nicotinic coenzymes (NAD^+, NADH, $NADP^+$, NADPH), glycosylated UDP-derivatives (UDP-galactose, UDP-glucose, UDP-N-acetyl-glucosamine, UDP-N-acetyl-galactosamine) reduced glutathione (GSH), nitrite and nitrate, purines and pyrimidines (hypoxanthine, xanthine, uric acid, guanosine, uracil, β-pseudouridine, uridine), were carried out using a Hypersil C-18, 250 x 4.6 mm, 5 µm particle size column, provided with its own guard column (Thermo Fisher Scientific, Rodano, Milan, Italy), following slight modifications of previously established ion pairing HPLC methods. The HPLC apparatus was a SpectraSYSTEM P4000 pump (Thermo Fisher Scientific) interfaced to a highly-sensitive UV6000LP diode array detector (Thermo Fisher Scientific), equipped with a 5 cm light path flow cell and set up between 200 and 300 nm wavelength[16,18]. The separation was carried out on 100 µl using a Hypersil 250 x 4.6 mm column, particle size 5 µm, equipped with its own guard column (Thermo Fisher Scientific, Rodano, Milan, Italy). A gradual gradient was formed from buffer A (10 mM tetrabutylammonium hydroxide, 10 mM KH2PO4, 0.125% methanol, pH 7.00) to buffer B (2.8 mM tetrabutylammonium hydroxide, 100 mM KH2PO4, 30% methanol , pH 5.5) as follows: 25 min. 100% buffer A; 8 min up to 80% buffer A; 10 min up to 70% buffer A; 12 min up to 55% of buffer A; 11 min up to 40% buffer A; 9 min up to 35% buffer A; 10 min up to 25% buffer A; 15 min 0% buffer A. A flow rate of 1.2 mL / min and a constant column temperature of 10 ° C were also used[16,18]. Assignments and calculations of the aforementioned compounds in cell extracts, were performed by comparing retention times, absorption spectra, and area of the peaks (calculated at 260 nm wavelength for all compounds but GSH, nitrite and nitrate that were calculated at 206 nm wavelength) of chromatographic runs of mixtures containing known concentrations of ultrapure standards.

Bibliography

16. Lazzarino, G.; Amorini, A.M.; Fazzina, G.; Vagnozzi, R.; Signoretti, S.; Donzelli, S.; Di Stasio, E.; Giardina, B.; Tavazzi, B. Single-sample preparation for simultaneous cellular redox and energy state determination. *Anal. Biochem.* **2003**, *322*, doi:10.1016/j.ab.2003.07.013.
18. Tibullo, D.; Giallongo, C.; Romano, A.; Vicario, N.; Barbato, A.; Puglisi, F.; Parenti, R.; Amorini, A.M.; Saab, M.W.; Tavazzi, B.; et al. Mitochondrial functions, energy metabolism and protein glycosylation are interconnected processes mediating resistance to bortezomib in multiple myeloma cells. *Biomolecules* **2020**, *10*, doi:10.3390/biom10050696.

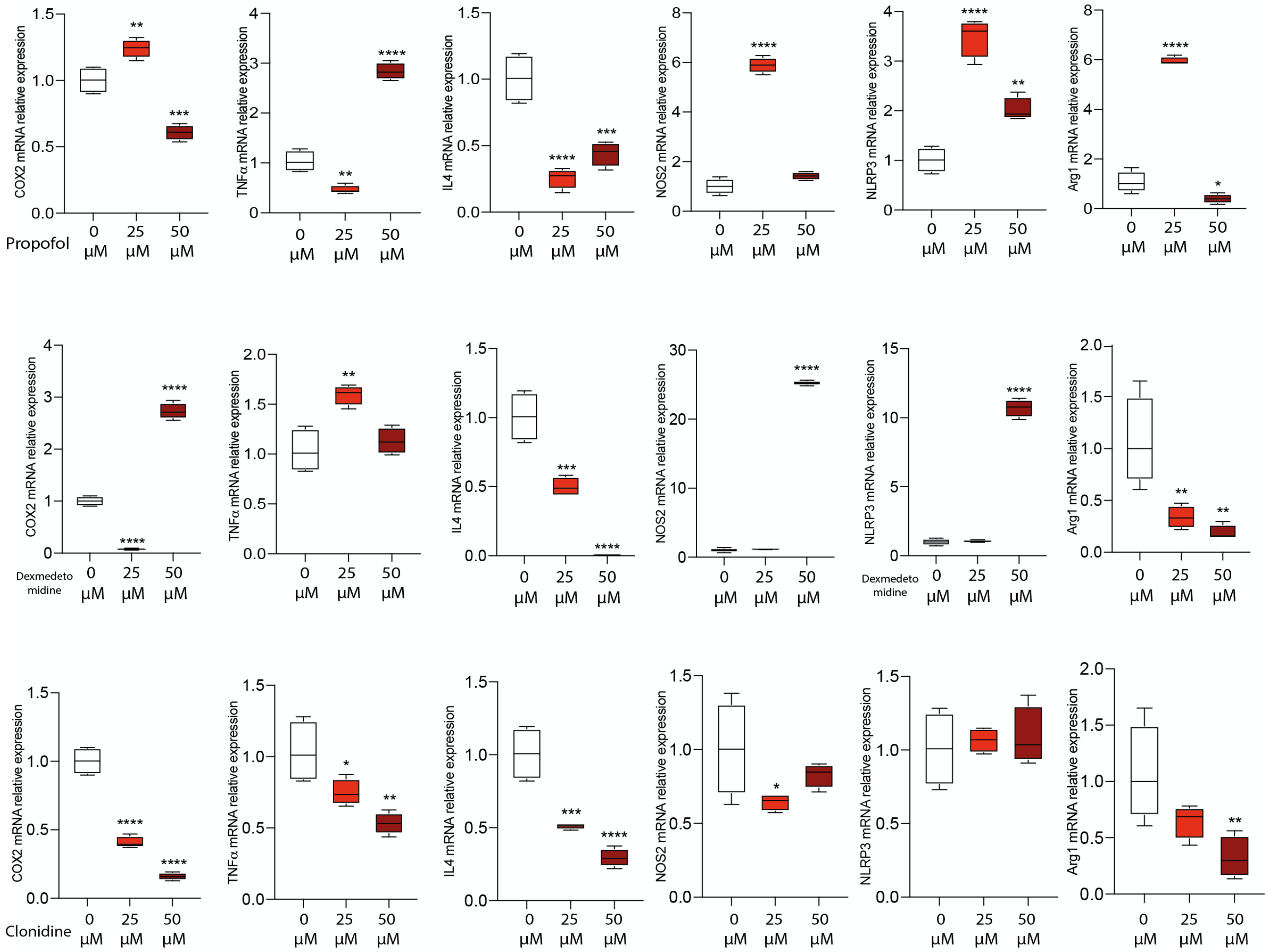

Figure S1: Effect of propofol, dexmedetomidine and clonidine treatment in the expression of COX2, TNF, IL4, NOS2, NLRP3 and ARG1 mRNA levels, under normoxic conditions.

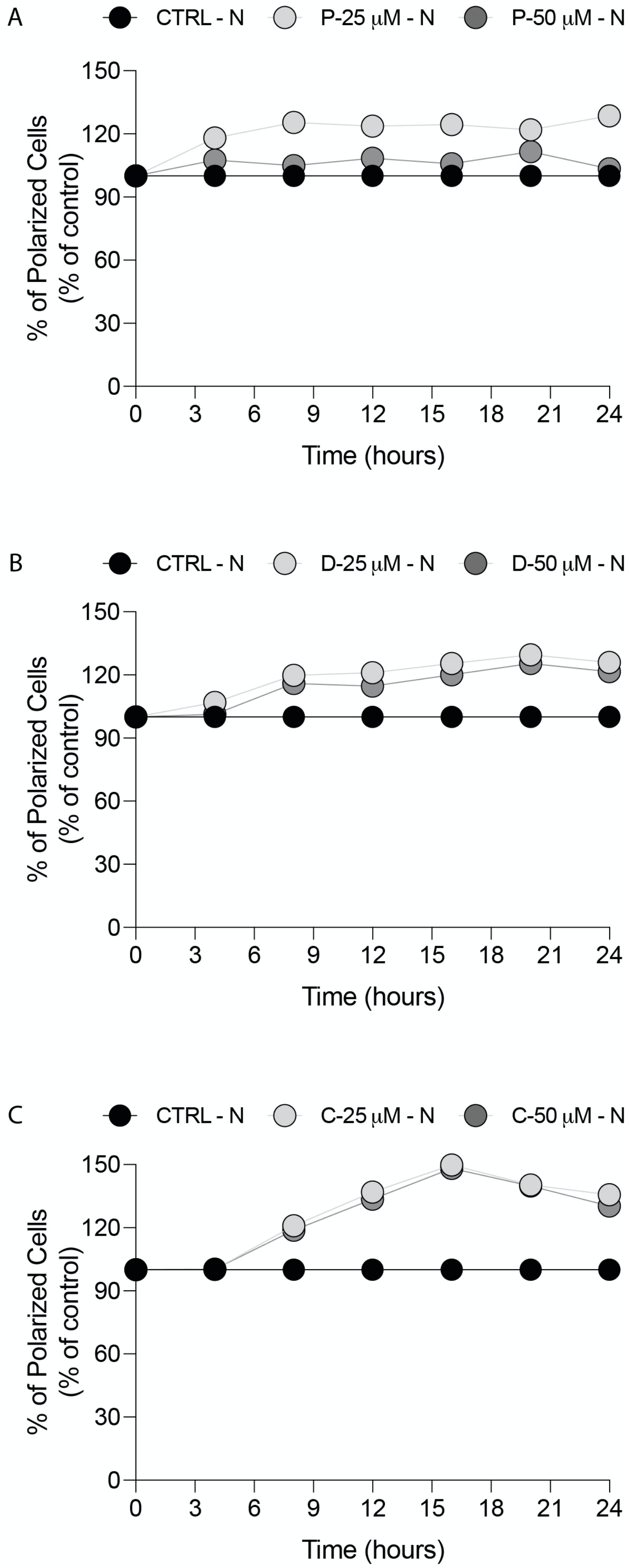

Figure S2: Real time analysis of ΔΨm modification following Propofol, dexmedetomidine and clonidine treatments under normoxic conditions.
